# Propagation of age‐related diseases due to the changes of lipid peroxide and antioxidant levels in elderly people: A narrative review

**DOI:** 10.1002/hsr2.650

**Published:** 2022-05-23

**Authors:** Julfikar Ali, Md. Abdul Aziz, Md. Mamun Or Rashid, Mohammad Anwarul Basher, Mohammad Safiqul Islam

**Affiliations:** ^1^ Department of Pharmacy, Faculty of Science Noakhali Science and Technology University Noakhali Bangladesh; ^2^ Laboratory of Pharmacogenomics and Molecular Biology, Department of Pharmacy Noakhali Science and Technology University Noakhali Bangladesh; ^3^ Department of Pharmacy, Faculty of Pharmacy and Health Sciences State University of Bangladesh Dhaka Bangladesh

**Keywords:** age‐related diseases, antioxidants, lipid peroxidation, oxidation, oxysterols, polyunsaturated fatty acid

## Abstract

**Background and Aims:**

Lipid peroxidation end products are the major culprit for inducing chronic diseases in elderly people. Along with the elevated level of lipid peroxide biomarkers, there is a significant disruption of antioxidants balance, which combinedly propagate the diseases of elderly people. The aim of the present review is to bridge the connection of changes in lipid peroxides biomarkers and antioxidants level with age‐associated diseases in elderly people.

**Methods:**

This narrative review was performed following a comprehensive search for suitable articles in multiple online databases, including PubMed, Google Scholar, EMBASE, Web of Science, Cochrane Library, and ScienceDirect using selected search terms. The most appropriate literature was included based on the selection criteria.

**Results:**

From the review, it is found that many age‐related diseases propagated with an increased level of the end products of lipid peroxide and reduced levels of antioxidants in elderly people. When the end products of lipid peroxidation increase in the body, it creates oxidative stress, which ultimately leads to many complicated diseases, including cancers, cardiovascular and neurogenic diseases, and many other chronic inflammatory diseases. The oxidative stress induced by peroxidation can be assessed by different lipid peroxide end products such as malondialdehyde, oxidized low‐density lipoprotein, isoprostanes, neuroprostanes, lipoperoxides, oxysterols (7‐ketocholesterol, 7β‐hydroxycholesterol), and many more.

**Conclusions:**

This study definitively answers the correlation between the changes in lipid peroxides and antioxidants level and age‐related diseases. Our narrative article recommends future investigations for elucidating the mechanisms rigorously to establish a compact correlation.

## 1 INTRODUCTION

Lipids are fundamental components of cell membranes that regulate both the function and structure of cells. Oxygen‐free radicals primarily target lipids and are responsible for the many pathological states of the body.^1^ Polyunsaturated fatty acids (PUFAs) (especially arachidonate) and those incorporated into lipids are the precursors of lipid peroxides. The concentration of lipid peroxides in our body is measured by biomarkers; some important lipid peroxidation biomarkers are acrolein, 4‐hydroxynonenal (4‐HNE), malondialdehyde (MDA), oxidized low‐density lipoprotein (LDL), isoprostanes, neuroprostanes, lipoperoxides, and oxysterols, including 7‐ketocholesterol (7‐KC) and 7β‐hydroxycholesterol (7β‐OHC).^2^, ^3^, ^4^ When the level of the biomarkers is elevated in the body, it creates many human diseases, including Alzheimer's disease (AD), cancer, cardiovascular diseases, immunological deficiency syndromes, and neurogenic disorders.^5^, ^6^

To identify the stage and severity of these diseases, some representatives of lipid peroxide biomarkers levels are used. They are isoprostanes, neuroprostanes, MDA, 4‐HNE, 7‐KC, and 7β‐OHC, and oxidized LDL are identified.^3^, ^4^ From various studies, evidence shows that during the aging process in both humans and animals model, lipid peroxidation levels increase whereas antioxidants levels decrease.^7^, ^8^ This disruption of antioxidants and lipid peroxides balance propagated diabetes and hypertension in elderly people.^8^ Another heart‐related disease is chronic pulmonary hypertension (PHT) which is caused by different factors, whereas lipid peroxides have a crucial part in vasoconstriction and structural remodeling. In PHT, an elevated level of isoprostanes is found in the patients when compared to healthy people.^9^ Along with the accumulation of various heart diseases, lipid peroxides also play a critical function in neurodegenerative disorders.^10^ In the AD brains, besides protein deposits aggregation as extracellular amyloid β (Aβ) plaques, evidence shows that reactive oxygen species (ROS) shows a vital role in the precipitation of nerve injury.^11^

The human brain has a large amount of easily peroxidizable fatty acids, and thus, it is particularly sensitive to the damage caused by lipid peroxidation.^12^, ^13^ Lipid peroxidation is also accountable for the pathophysiology of another neurogenic disorder, Parkinson's disease (PD). Pathophysiological damage of PD usually starts before the symptoms appear in the body. After 80% of the dopaminergic neurons are damaged in the substantia nigra pars compacta (SNpc), PD symptoms start to appear. At the same time, patients remain unaware of the diseases. However, the exact etiology of PD is still obscure. One of the major causes of PD is thought to be the oxidative stress (OS) of dopaminergic neurons in SNpc. Furthermore, studies revealed that lipid peroxidation reaction results as a consequence of OS. Studies found that the plasma MDA levels increased by two times in the PD patients when compared with the control population.^14^ Among the different pathologies of lipid peroxidation reaction in the human body, cancer is another incident that happens from lipid peroxidation reaction.^15^, ^16^, ^17^

Lipid peroxidation was found to be involved in the progression of colorectal cancer. Generation of lipid peroxidation end products such as 4‐HNE and MDA create proteins cross‐linked with DNA. As a result, damage to DNA and cell membrane occurs. Moreover, the concentration of MDA and 4‐HNE has been demonstrated to increase significantly following the clinical staging of colorectal cancer tissue.^18^ Apart from colorectal cancer, breast, lung, and many more malignancies are associated with the lipid peroxides elevation in the body.^19^ From the above evidence, it is clear that age‐associated diseases are precipitated from increased lipid peroxidation end products in the body.

## 2 LIPID PEROXIDATION REACTION

Trienoic linolenic acid is a common PUFA present in cell membrane lipids that undergoes autoxidation. Lipid peroxidation reaction usually consists of three steps: Initiation followed by propagation and termination reactions. In the initiation step, due to the presence of strong oxidants, an alkyl free radical (L^•^) of the PUFA is formed through the abstraction of a weakly bonded allylic hydrogen.

### 2.1 Initiation

In^•^ + L‐H → L^•^ + In‐H

[In, initiator; L‐H, fatty acid; L^•^, alkyl radical]

Later, the alkyl free radical undergoes an addition reaction in the presence of oxygen that produces another conjugated peroxyl free radical in the body. This peroxyl radical abstracts another hydrogen from a second PUFA that generates a hydroperoxide and a different alkyl free radical.

### 2.2 Propagation

O_2_ + L^•^ → l‐OO^•^ → l‐OOH + L^•^

[L‐OO, conjugated peroxyl radical; l‐OOH, hydroperoxide]

This cycle is called the propagation reaction phase of peroxidation. Termination of lipid peroxides occurs via the involvement of two alkyl free radicals, two peroxyl free radicals, or their combinations.^1^, ^20^

### 2.3 Termination

2 l‐OO^•^ → l‐OO‐OO‐L → NRP + O_2_

[NRP, nonradical products]

Lipid peroxides are toxic in nature and may damage most body cells.^5^ In the reaction process, highly reactive hydroxyl radicals (^•^OH) are derived from high‐energy sources of irradiation (aqueous solutions), which then attack and disturb the majority of the biological molecules, including lipids of the cell membrane. Lipid peroxidation reaction also involves transition metal ions. When Cu^+2^, Fe^2+^, or chelates of such ions like Fe^2+^‐ADP are added to isolated biological membranes or liposomes, peroxidation occurs. The measurement of lipid peroxidation level is usually carried out by the thiobarbituric acid (TBA) test, high‐performance liquid chromatography (HPLC), gas chromatography–mass spectrometry (GC‐MS), and liquid chromatography–mass spectrometry (LC–MS).^21^, ^22^ Among the different assays, the batch spectrofluorometric and spectrophotometric TBA‐dependent techniques are the most frequently applied to determine the extent of lipid peroxidation.^22^

## 3 BIOMARKERS OF LIPID PEROXIDES

In general, lipid peroxides biomarkers are hydroperoxides, hydroxides, isoprostanes, neuroprostanes, MDA, 4‐HNE conjugated diene, ethane, pentane, aldehydes, ketones, 7‐KC, and 7β‐OHC, oxidized LDL, and so forth.^3^ Among them, the most common and easily detectable lipid peroxidation biomarkers are MDA, 4‐HNE, 7‐KC, and 7β‐OHC, and isoprostanes.^4^, ^23^, ^24^, ^25^ From the end products of lipid peroxides, hydroperoxides are primarily generated from PUFA and cholesterol. Hydroperoxide products are naturally unstable and commonly evaluated as hydroxides following reduction reaction. The second notable peroxidation end products are hydroxides, readily available in biological specimens. Isoprostanes (IsoP) is a series of compounds similar to prostaglandins that are generated by the peroxidation of lipids, mainly arachidonic acids independent of enzymes such as cyclooxygenase. Arachidonic acid oxidation reaction yields A2, D2, E2, F2, and J2‐IsoPs. From these IsoPs, F2s have been studied most extensively.^26^ Thiobarbituric acid reactive substances (TBARS) and MDA have commonly been considered as classical lipid peroxidation biomarkers for a long time for their reduced cost and simple nature. Previous research have demonstrated the proatherogenic and proinflammatory activities of oxidized LDL (oxLDL). For that reason, the circulating level of oxLDL has been a cornerstone of atherosclerosis and cardiovascular disease‐related biomarker analysis.^3^

The end products of the peroxidation of lipids are simpler to identify and more accurate compared to the identification of ROS, RNS (reactive nitrogen species), and related oxidation products.^27^ Lipid peroxide products can be identified through the application of several probes and techniques. The most commonly applied probes and techniques are chemiluminescence probes, fluorescence probes, and electron spin resonance (ESR) spin trapping method. Again, to detect and identify the complex mixtures, including IsoPs and NPs, more powerful tools are used. Some of these tools are matrix‐assisted laser desorption and ionization time‐of‐flight mass spectrometry or ion‐spray mass spectrometry and electrospray ionization. The oxidation products of linoleates are detected by the application of GC‐MS and HPLC which provides greater accuracy and sensitivity.^27^ HPLC is a vital technique to determine another product of lipid peroxidation, MDA. This method has advantages in terms of recovery, specificity, and reproducibility.^23^

## 4 RELATIONSHIP BETWEEN LIPID PEROXIDE AND DISEASES IN ELDERLY PEOPLE

According to the accumulating evidence, lipid peroxidation end products are responsible for the cellular and tissue dysfunction in elderly people that leads to age‐related diseases. Brain, heart, and skeletal tissues are some kinds of tissues or organs where the turnover of cells is lower or sometimes absent. As a consequence, a large amount of lipid peroxidation end products accumulates in these tissues and organs that primarily act as physiologic mediators. Following the continuous aggregation of these products, numerous age‐associated diseases in the elderly population progress, including cancers, cardiovascular disorders, diabetes and related complications, endocrine and metabolic diseases, neurological disorders, and other oxidative stress‐associated conditions.^7^ As a consequence of the peroxidation of membrane phospholipids, cell membrane permeability increase, membrane integrity decreased, DNA was damaged, and cell death occurred.^28^ The most common end products of lipid peroxidation bind with DNA and produce DNA adducts which are toxic in nature and get accumulated in the liver, colon, pancreas, white blood cells, and breast in healthy humans that ultimately result in organ‐related disease in the long run.^29^ 8‐hydroxydeoxyguanosine (8‐OHdG) is a well‐known biomarker of oxidative stress‐related DNA damage. Lipid peroxides bind with DNA and produce 8‐OHdG. Increased level of 8‐OHdG expression in certain tissues of the body has been found to be associated with several chronic diseases of the liver.^30^

The effect of peroxidation on the cell membrane lipids is even more harmful, and it hampers the bilayer structure of the membranes and modifies its characteristic features, for example, membrane permeability, fluidity, and thickness of lipid bilayer. The increased permeability of the membrane disrupts the ionic gradients that attenuate the metabolic system.^31^ Acrolein, a carcinogenic pollutant, and lipid peroxidation product is found ubiquitously in the biological systems as well as in the environment. Acrolein bound to the protein and altered the structure and activity of the bounded proteins. Acrolein binds with nucleophiles, specifically the amino, imidazole, and sulfhydryl group of lysine, cysteine, and histidine, respectively producing an acrolein‐amino acid adduct that further affects a carbonyl group of the proteins and leads to cell death.^32^ In addition to oxidatively modified DNA, lipids, nucleic acids, and proteins, the disruption of antioxidant balance biomarkers is also considered for oxidative stress.^33^ This disrupted balance causes many human morbidities and mortalities. However, if the dietary or supplemented antioxidants reduced the oxidative stress‐induced diseases is not clear yet. However, recent studies on antioxidants as a dietary approach to reduce oxidative stress‐induced damage found that many chronic and degenerative diseases ameliorate, and thus antioxidant therapy represents a promising avenue for treatment.^34^

**Figure 1:**
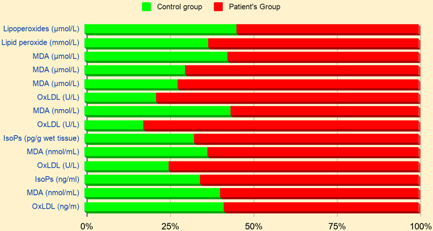
Comparison of lipid peroxide biomarker levels in elderly patients and healthy controls.

The alteration of lipid peroxide products and their involvement with age‐associated diseases are shown in Table 1. From examining Table 1, we may find that biomarkers of lipid peroxides are involved in many elderly diseases. Compared to the controls, the patient group shows an elevated MDA level, oxLDL, and IsoPs in AD, cancers, cardiovascular diseases, metabolic disorders, and PD which is depicted in Figure 1.

**Table 1:** Change of lipid peroxide biomarkers level in diseases of elderly people

Biomarkers	Value in controls	Value in patients	No. of patients	Age (years)	Findings	Diseases	References
Lipoperoxides (μmol/L)	3.65 (3.29–3.89)	4.37 (3.85–5.75)	50	<70	↑ Lipoperoxides	Ischemic heart disease	[35]
Lipoperoxides (μmol/L)	3.65 (3.29–3.89)	4.37 (3.85–5.75)	50	<70	↑ Lipoperoxides	Peripheral arterial disease	[35]
Lipid peroxide (mmol/L)	1.00 ± 0.12	1.71 ± 0.38	30	59.1 ± 11.7	↑ Lipid peroxide	Stroke	[36]
MDA (μmol/L)	0.3 ± 0.009	0.4 ± 0.007	80	39.3 ± 0.5	↑ MDA	Diabetes 2	[37]
MDA (μmol/L)	2.72 ± 0.13	6.33 ± 0.33	26	50±	↑ MDA	Breast cancer	[38]
MDA (μmol/L)	2.26 ± 0.14	5.87 ± 0.29	12	N/A	↑ MDA	Lung cancer	[38]
OxLDL (U/L)	78.8 ± 56.1	288.3 ± 262.3	17	53–70	↑ OxLDL	Breast cancer	[39]
MDA (nmol/L)	6.73 ± 2.66	8.7 ± 2.99	42	49.6 ± 8.22	↑ MDA	Ovarian cancer	[40]
OxLDL (U/L)	78.8 ± 56.1	367 ± 524	15	53–70	↑ OxLDL	Ovarian cancer	[39]
IsoPs (pg/g wet tissue)	200 (81–260)	410 (240–880)	19	79 ± 2.1	↑ IsoPs	Alzheimer's	[41]
MDA (nmol/ml)	9.38 ± 5.69	16.11 ± 7.16	62	73.3 ± 6.8	↑ MDA	Alzheimer's	[42]
OxLDL (U/L)	40.69 ± 2.27	120.9 ± 18.4	5	76	↑ OxLDL	Alzheimer's	[43]
IsoPs (ng/ml)	0.36	0.68	61	64 ± 6	↑ IsoPs	Parkinson's	[44]
MDA (nmol/ml)	5.1 ± 1.26	7.48 ± 1.55	80	57.5 ± 12.1	↑ MDA	Parkinson's	[45]
OxLDL (ng/ml)	24.7 ± 5.7	34.7 ± 12.2	45	63.6 ± 13.5	↑ OxLDL	Parkinson's	[46]

Abbreviations: LDL, low‐density lipoprotein; MDA, malondialdehyde; OxLDL, oxidized LDL.

## 5 LIPID PEROXIDATION AND CARDIOVASCULAR DISEASES

Oxidation of lipid molecules is catalyzed by different enzymatic systems. Various enzymes, in particular, cyclooxygenases (COXs), cytochrome P450 (CYP) monooxygenases, and lipoxygenases (LOXs) are responsible for the oxidation of lipids molecule and produces a large number of potent bioactive metabolites, such as eicosanoids, docosanoids, and octadecanoid. These metabolites are involved in several biochemical and signaling pathways.^47^ Eicosanoids are the products of PUFAs such as arachidonic, dihomo‐γ‐linolenic, and eicosapentaenoic acids. They are also the most studied class of bioactive lipids, which have an important role in inflammation. COXs enzymes catalyze the conversion of eicosanoids to prostanoids, which are a subclass consisting of prostacyclins, prostaglandins (PGs), and thromboxanes (TXs) family. These metabolites are involved in inflammation regulation, pain reception, adipogenesis, immune modulation, vessel tone, permeability control, and platelet aggregation. In atherosclerotic and ischemic heart disease treatment, the COX pathway is considered one of the major targets.^48^

Metabolites of the LOX isoenzymes mainly regulate vascular permeability and affect epithelial barrier function. A large member of the CYP multigene superfamily converts omega‐3 and omega‐6 fatty acids into CYP eicosanoids, which are bioactive lipid mediators.^49^ CYP enzymes produce different epoxy and/or hydroxy metabolites, where CYP2C and CYP2J enzymes produce the epoxygenases and CYP4A and CYP4F produce the hydroxylases of PUFA. Depending on the type and concentration of these metabolites, numerous physiological and pathophysiological changes occur. The changes in CYP eicosanoid products have been correlated with the progression and development of cardiac hypertrophy, hypertension, inflammatory disorders, myocardial infarction, and stroke.^50^ In ischemic brain injury, oxidative stress has been observed to play a critical role. Additionally, to the high oxidation level of PUFA in the brain, poor activity of catalases, glutathione peroxidases, and superoxide dismutase (SODs) is also observed. Furthermore, increased homocysteine concentration is also found in the brain, which is responsible for the enhanced oxidative stress leading to acute thrombotic stroke.^36^, ^51^ From the above, it is concluded that any complicated heart diseases are propagated from the elevation of lipid peroxides molecules and the disruption of antioxidant balance.

## 6 LIPID PEROXIDATION AND NEUROGENIC DISORDERS

The end products of lipid peroxidation are highly associated with multiple neurogenic disorders such as AD, HD (Huntington's disease), and PD. Evolving research continues to increase in relation to lipid peroxidation and neurodegenerative diseases.^52^ Different parts of the brain, including the entorhinal and frontal cortex, hippocampus, and associated brain structures, are involved in the cognition amygdala, prefrontal cortex, hypothalamus, and other regions of the brain's emotional behaviors. AD is described by neuron degeneration in those regions of the brain. It is believed that abnormal production and aggregation of amyloid β‐peptide (Aβ) is a pivotal reason in AD propagation.^53^ A common lipid peroxidation end product, 4‐HNE, is involved in the suppression of Aβ, which is demonstrated to be linked with the progression of AD. In contrast, 4‐HNE is associated with γ‐secretase enzyme inhibition, directly correlated to the synthesis of insoluble Aβ peptides.^54^

α‐synuclein is another protein responsible for the aggravation of PD, and the core function of α‐synuclein is mitochondrial function and formation of synaptic vesicles.^55^, ^56^ Acrolein, a lipid‐peroxidation product, also modifies the activity of α‐synuclein in a dopaminergic neuronal system that results in mitochondrial dysfunction.^57^ The accumulation and modification of these substances are the primary components of Lewy bodies, mainly found in the substantia nigra and putamen. These areas in the brain are mainly responsible for learning capacity and motor skills. Dopamine, the major neurotransmitter associated with controlling motor function, is observed to be significantly reduced in neurons, leading to neuronal destruction or death. Apart from the Lewy bodies, mutations in certain genes such as ubiquitin and parkin carboxyl‐terminal hydroxylase could be linked to disease progression and development.^58^ It is well proven that 4‐HNE and MDA adducts are related to the increased level of Lewy bodies in the brain stem neurons and neocortical.^59^, ^60^ 4‐HNE is related to the alteration of the dopamine transport pathway.^61^ These observations suggest that the loss of dopamine causes PD.^52^ Apart from AD and PD, lipid peroxides have a crucial activity in developing HD and multiple sclerosis.^62^, ^63^

## 7 LIPID PEROXIDATION AND CANCER

Accumulating studies have shown that lipid peroxidation end products, particularly 4‐HNE, are also a notable biomarker of oxidative stress that has been evaluated in different cancer cells.^64^ Many clinical studies found that an elevated plasma MDA level is responsible for benign breast disease and malignant breast tumors compared with controls.^65^ Lipid peroxidation products have been involved in both the progression and the development of cancer. The capacity of end products of lipid peroxides in the DNA damage mechanisms has been examined in biological systems and solutions. Previous reports established a relation between clastogenic factors and lipid peroxidation process in particular biological specimens which are associated with the chromosomal breakage.^66^ Clastogenic factors induce DNA single‐strand breaks with high efficiency. MDA is well known for its ability to cause polymerization and crosslinking of macromolecules and react with DNA. Generally, peroxidative degradation products of PUFAs may either initiate the process or act as a promoter, thus favoring tumor growth.^67^ The impact of lipid peroxidation in breast malignancy is unclear; surprisingly, some studies favor that lipid peroxidation shows the anticarcinogenic mechanism in breast cancer despite inducing it.^68^ But there was a positive association of lung cancer found with lipid peroxidation. As the stage of lung cancer progressed, lipid peroxides elevated.^69^

## 8 OXYSTEROLS IN ELDERLY DISEASES

Oxysterols are generally lipid peroxidation products, more specifically, cholesterol metabolites that are generated via enzymatic or nonenzymatic (radical) pathways.^70^ Some of the notable oxysterols are 7‐KC, 7α‐hydroxycholesterol (7α‐OHC), 7β‐OHC, 25‐hydroxycholesterol (25‐OHC), 27‐hydroxycholesterol (27‐OHC), 5α,6α‐epoxycholesterol (5α,6α‐EC), 5β,6β‐epoxycholesterol (5β,6β‐EC), and cholestan‐3β,5α,6β‐triol, all produced by autoxidation of cholesterols.^71^ Oxysterols have been studied extensively for their role in vascular aging due to a few crucial properties such as proapoptosis, proinflammation, and pro‐oxidation. Increasing evidence showed the impact of different oxysterols in the age‐associated intimal stiffening, endothelial dysfunction, smooth muscle cell migration, and arterial thickening.^72^, ^73^

Elderly diseases such as cardiovascular diseases and neurogenic disorders (AD, PD) are correlated with an increased level of 7‐KC and 7β‐OHC, which cause oxiapoptophagy, one type of cytotoxic activity.^74^ 7‐KC is one of the most concentrated oxysterols and unlike cholesterols, it continuously shows cytotoxicity leading to cardiovascular events such as heart failure. Oxysterols can easily pass the blood−brain barrier, deposit in brain tissues, and cause neurodegenerative disorders, including AD and PD.^75^ It is proved by the line of evidence that elevated oxysterols level, particularly 7‐KC and 7β‐OHC, or reduced level of 27‐OHC, may lead to neurodegenerative diseases.^76^ However, there is a toxicological concern for a few oxysterols in cholesterol‐containing food products (milk, skimmed milk powder) that require routine analysis. While oxysterols such as 25‐OHC and 27‐OHC have shown notable broad‐spectrum antiviral actions, only their toxicologic properties have been demonstrated so far for radically produced oxysterols when present excessively in the human body and ingested foods (milk‐derived products like chocolate).^76^, ^77^ So far, the toxicity of two common oxysterols, 7β‐OHC and 7‐KC, has been analyzed in the heart, brain, and gut, showing oxiapoptophagy leading to major elderly diseases, including cardiovascular disease and cancer, diabetes, and aging.^71^

## 9 CHANGES IN ANTIOXIDANTS LEVEL IN ELDERLY DISEASES

Antioxidant molecules are known as free radical scavengers that have a prominent action in multiple age‐dependent diseases. It is already established that antioxidants prevent many age‐related diseases like AD, coronary heart disease (CHD), cancers, PD, and related chronic inflammatory diseases.^78^ Past research has demonstrated that diabetes patients with a smoking history have a significantly higher level of lipid peroxides and a significantly lower level of vitamin C and β‐carotene.^79^ The latest research reports that patients with AD have significantly reduced levels of vitamin A, C, and E, α and β‐carotene, lutein, lycopene, and uric acid in their plasma.^80^ Another study reported that the depletion of the most antioxidant defense enzymes such as GPx, glutathione S‐transferase (GST), and SOD could result in AD or mild cognitive impairment.^81^ Venkateshappa et al.^82^ have found that a significantly reduced level of catalase, GSH, GSH reductase (GR), GPx, and SOD with increasing age lead to the development of PD. Kim and co‐workers also revealed a significantly decreased concentration of zinc (Zn) and selenium (Se) in patients with cancer in comparison to the control population. Serum GPx level was also reduced in cancer patients than in the controls.^83^ The changes in antioxidants levels in elderly diseases are shown in Table 2.

**Table 2:** Changes of antioxidants level in diseases of elderly people

Biomarkers	Value in control	Value in patients	No. of patients	Age (years)	Findings	Diseases	References
GSH (μM)	1496 ± 365	661 ± 276	112	48 ± 10	↓ GSH	Breast cancer	[84]
GSH (nmol/ml)	47.4 ± 29.7	43.4 ± 21.4	62	73.3 ± 6.8	↓ GSH	Alzheimer's	[42]
SOD (U/gHb)	1120 ± 50	1510 ± 90	27	72.3 ± 6.5	↑ SOD	Alzheimer's	[85]
SOD (U/ml)	800 ± 200	830 ± 320	30	59.1 ± 11.7	↑ SOD	Stroke	[36]
Plasma SOD (U/µl)	2580 ± 1170	3680 ± 1270	48	64.9 ± 9.5	↑ SOD	Parkinson's	[86]
GPx^+^(cells/mm^2^)	282 (201−417)	515 (306−750)	4	65.5 ± 8.6	↑ GPx^+^	Parkinson's	[87]

Abbreviation: SOD, superoxide dismutase.

**Figure 2:**
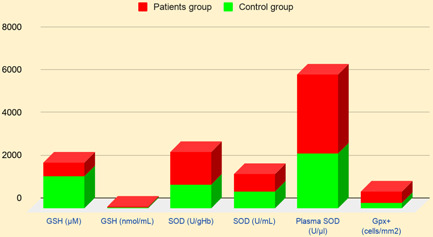
Comparison of antioxidant levels in elderly patients and healthy controls.

It is observed that SOD, GPx^+^ levels are significantly higher in cancer and neurogenic patients, and the level of GSH is low. An increased concentration of plasma SOD results from the enhanced concentration of extracellular SOD and greater activity of SOD is relative to greater SOD protein.^86^ In the AD group, SOD level was found to increase where the melatonin level found decreased, indicating the disrupted balance between oxidant and antioxidant systems,^85^ illustrated in Figure 2.

## 10 ANTIOXIDANT THERAPY FOR AGE‐ASSOCIATED DISEASES

Numerous investigations show that antioxidant molecules inhibit the oxidation process and delay or prevent oxidative stress‐induced diseases such as cancer, cardiovascular diseases, neurogenic disorders, and related chronic diseases responsible for a significant amount of deaths in today's world. Intake of high vitamin C, vitamin E, and flavonoids decrease the risk of CHD. Again, intake of micronutrients, including antioxidants β‐carotene, vitamin C and E, and Se are involved in decreased risk of cancer.^88^ Previous studies revealed a potential relationship between high intake of α‐lipoic acid, vitamin E, or Se and reduced diabetic neuropathy in patients with both Type 1 and Type 2 diabetes.^89^ To prevent or reduce oxidative stress‐dependent diseases, cells maintain a balance in the production of oxygen radicals and in the removal of them by an antioxidant defense mechanism. The defensive antioxidants enzymatic molecules include SOD, GPx, catalase, and GST. Among them, catalase and SOD reduce hydrogen peroxide and superoxide anion, respectively. Again, GPx reduces glutathione which ultimately reduces hydrogen peroxides. GST, on the other hand, causes the metabolism of xenobiotic compounds by the formation of GSH conjugates with them.^90^

## 11 METHODOLOGY

This narrative review was performed following a comprehensive search for suitable articles in multiple online databases, including PubMed (https://pubmed.ncbi.nlm.nih.gov/), Google Scholar (http://scholar.google.com/), EMBASE (https://www.embase.com/), Web of Science (https://mjl.clarivate.com/), Cochrane Library (https://www.cochranelibrary.com/), and ScienceDirect (https://www.sciencedirect.com/). We have applied the following search terms either alone or in combination: “lipid peroxidation,” “antioxidants,” “age‐related diseases,” “oxidation,” “polyunsaturated fatty acid,” “lipid peroxide and elderly disease,” “lipid peroxide and cardiovascular disease,” “lipid peroxide and neurogenic disorders,” “lipid peroxide and cancer,” “polyunsaturated fatty acid,” and “biomarkers of lipid peroxides.”

We have included the articles that discussed (a) the lipid peroxidation mechanism, (b) the role of lipid peroxides in elderly diseases, cardiovascular diseases, neurogenic disorders, and cancer, (c) the role of antioxidants in elderly diseases, and (d) antioxidant therapy in age‐related diseases. Besides, some articles were excluded in our study for being (a) letter to editor, (b) commentaries, or (c) not providing enough data to be included. For the statistical analyses, we have applied IBM SPSS software package (version 25).

## 12 CONCLUSION AND FUTURE PERSPECTIVES

Lipid peroxidation products are highly reactive compounds that can modify macromolecules, including nucleic acids, phospholipids, leading to lipoxidation. Lipid peroxidation end products induce oxidative stress and altered antioxidant levels play a vital role in the progression of age‐associated diseases. Lipid peroxidation products have been associated with cardiac hypertrophy, contractile dysfunction apoptosis, and thus, playing a role in heart failure. The lipid peroxidation biomarkers have been observed to be associated with several neurogenic disorders, including AD, PD, HD, and amyotrophic lateral sclerosis.

In the field of age‐associated diseases, the association of lipid peroxidation products with these diseases will continuously be investigated. Imbalanced metabolism of energy, altered levels of antioxidants, and dysfunction of mitochondria have been observed in several neurogenic diseases leading to oxidative stress, progressive memory loss, and physical impairments. 4‐HNE plays an essential pathological role in cancer and macromolecular adducts of 4‐HNE found in the mitochondria are correlated with the development of cancer through the modulation of mitochondrial function. Oxysterols such as 7‐KC and 7β‐OHC were also found to be correlated with the pathogenesis of cardiovascular diseases, cancers, AD, PD, and aging. Antioxidant therapies have been considered a promising strategy to treat oxidative stress‐induced diseases lowering lipid peroxide product levels. From the studies' analysis, it is well understood that increased concentration of lipid peroxide products and decreased concentration of antioxidants are associated with elderly diseases.

Although many questions related to age‐related disorders and lipid peroxides or antioxidants are yet to be elucidated, it is apparent that an imbalance in lipid peroxide end products or antioxidants has an imperative function in maintaining the aging process. Besides, particular lipids and lipid‐related biomarkers have been reported to enhance or diminish in an age‐associated manner. Future research should aim to elucidate the underlying mechanisms targeting lifespan alteration in different model organisms. This would lead to discovering molecular targets and possible treatments that could be used for elongating the human lifespan. Antioxidant supplements can compensate for the reduced level of antioxidants and lower lipid peroxide end‐product levels to prevent chronic diseases and ultimately act as a therapy to aid elderly conditions. However, further studies are necessary to consider antioxidant therapy for chronic inflammatory age‐related diseases.

## AUTHOR CONTRIBUTIONS

*Conceptualization*: Md. Mamun Or Rashid and Mohammad Safiqul Islam. *Writing—original draft*: Julfikar Ali and Md. Abdul Aziz. *Formal analysis*: Mohammad Anwarul Basher. *Writing—review and editing*: Md. Abdul Aziz, Julfikar Ali, and Mohammad Safiqul Islam. All authors have read and approved the final version of the manuscript. The corresponding author had full access to all the data in this study and takes complete responsibility for the integrity of the data and the accuracy of the data analysis.

## CONFLICTS OF INTEREST

The authors declare no conflicts of interest.

## TRANSPARENCY STATEMENT

The corresponding author affirms that this manuscript is an honest, accurate, and transparent account of the study being reported; that no important aspects of the study have been omitted; and that any discrepancies from the study as planned (and, if relevant, registered) have been explained.

## Data Availability

The data that support the findings of this study are available on request from the corresponding author.
